# A high-resolution method for the localization of proanthocyanidins in plant tissues

**DOI:** 10.1186/1746-4811-7-13

**Published:** 2011-05-20

**Authors:** Shamila W Abeynayake, Stephen Panter, Aidyn Mouradov, German Spangenberg

**Affiliations:** 1 Department of Primary Industries, Biosciences Research Division, Victorian AgriBiosciences Centre, 1 Park Drive, Bundoora, Victoria, 3083, Australia; 2 Molecular Plant Breeding Co-operative Research Centre, 1 Park Drive, Bundoora, Victoria, 3083, Australia; 3 La Trobe University, Bundoora, Victoria, 3083, Australia

## Abstract

**Background:**

Histochemical staining of plant tissues with 4-dimethylaminocinnamaldehyde (DMACA) or vanillin-HCl is widely used to characterize spatial patterns of proanthocyanidin accumulation in plant tissues. These methods are limited in their ability to allow high-resolution imaging of proanthocyanidin deposits.

**Results:**

Tissue embedding techniques were used in combination with DMACA staining to analyze the accumulation of proanthocyanidins in *Lotus corniculatus *(L.) and *Trifolium repens *(L.) tissues. Embedding of plant tissues in LR White or paraffin matrices, with or without DMACA staining, preserved the physical integrity of the plant tissues, allowing high-resolution imaging that facilitated cell-specific localization of proanthocyanidins. A brown coloration was seen in proanthocyanidin-producing cells when plant tissues were embedded without DMACA staining and this was likely to have been due to non-enzymatic oxidation of proanthocyanidins and the formation of colored semiquinones and quinones.

**Conclusions:**

This paper presents a simple, high-resolution method for analysis of proanthocyanidin accumulation in organs, tissues and cells of two plant species with different patterns of proanthocyanidin accumulation, namely *Lotus corniculatus *(birdsfoot trefoil) and *Trifolium repens *(white clover). This technique was used to characterize cell type-specific patterns of proanthocyanidin accumulation in white clover flowers at different stages of development.

## Background

Proanthocyanidins, or condensed tannins, are polymers of flavan-3-ol subunits, which are produced by the flavonoid secondary pathway in many plants. Proanthocyanidins are best known for their protein-binding ability and are commercially significant because of their antioxidant properties and their potential health benefits when included at a low level in the diets of humans and livestock [1-5]. Proanthocyanidins are produced naturally in the leaves, flowers, fruit, seeds, bark and roots of many plant species [6-11]. A number of quantitative methods have been developed to analyze the level and subunit composition of proanthocyanidins in bulk tissue samples [5,7,12-14]. These methods can provide information about the degree of polymerisation and the hydroxylation pattern and stereochemistry of flavan-3-ol subunits.

Vanillin and 4-dimethylaminocinnamaldehyde (DMACA) are commonly used for histochemical staining of proanthocyanidins and their immediate precursor molecules, namely, flavan-3,4-diols and flavan-3-ols, in fresh plant material [15-20]. DMACA reagent stains proanthocyanidins a blue color by binding to *meta*-oriented dihydroxy- or trihydroxy-substituted benzene rings [14,19,21]. The main disadvantage of histochemical staining of fresh plant tissues is that cellular integrity is compromised during the sectioning process.

A range of methods have been used to localize proanthocyanidins, but each has limitations that need to be considered when planning an experiment. Established methods for localization of proanthocyanidins using electron microscopy [22-25] require a high level of technical expertise and are expensive. Epoxy and glycolmethacrylate resins have been used as embedding media for plant tissues prior to sectioning and staining to visualize proanthocyanidins [16,24,26]. Staining of plant tissues embedded in epoxy resin with Sudan Black to detect proanthocyanidin deposits also stained lipid bodies [24]. Staining of sections from samples embedded in glycolmethacrylate resin has been used in combination with the more specific DMACA staining reagent to preserve the fine structure of plant tissues [16]. This method involved heating of glycolmethacrylate-embedded semithin sections in a microwave oven in the presence of a staining solution containing DMACA to enhance the staining process. However, not all cells in sections were fixed equally well, suggesting that the glutaraldehyde fixative had not penetrated the tissue sufficiently. The heating time also needed to be carefully controlled to avoid discoloration and each section had to be treated with fresh staining solution, which was deactivated upon heating. Proanthocyanidins have also been visualized by fixing fresh samples in a solution of formalin and ferrous sulphate, but this method also stains other phenolic substances [27].

Specific staining for proanthocyanidins, flavan-3-ols and flavan-3,4-diols without damage to the fine structure of plant tissues is a challenging task, due to the acidity of the DMACA staining solution [27,28]. In this study, DMACA staining was used in combination with two commonly-used embedding techniques to analyze the accumulation of proanthocyanidins in *Lotus corniculatus *and *Trifolium repens *tissues. Physical integrity of plant tissues was retained during the staining, fixing, embedding and sectioning steps. Embedding of the tissues in LR White and paraffin matrices lead to the appearance of brown coloration in proanthocyanidin-accumulating cells. This is likely to have been the result of non-enzymatic oxidation of proanthocyanidins and the formation of colored semiquinones and quinones. This method is very simple and has the potential to provide high-resolution images showing cell-specific localization of proanthocyanidins in a range of plant tissues.

## Results and discussion

### Proanthocyanidin accumulation in *Lotus corniculatus *and *Trifolium repens*

**Figure 1:**
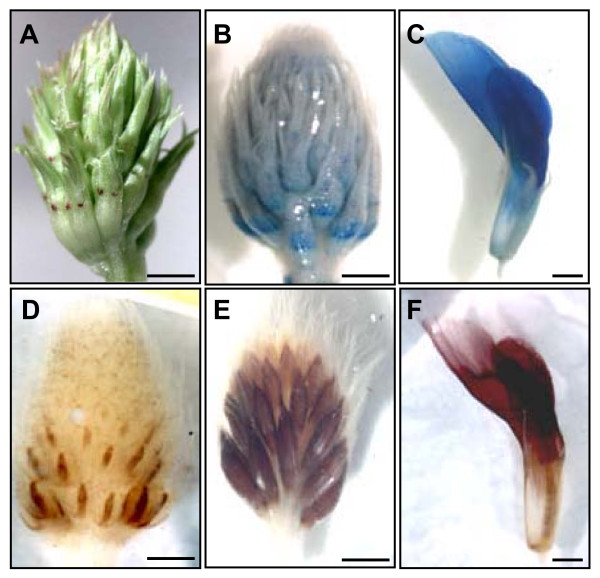
**Proanthocyanidin accumulation in white clover tissues**. An immature inflorescence before (A) and after (B) DMACA staining. A mature flower after DMACA staining (C). Immature inflorescences at early (D) and late (E) stages of development and a mature flower (F) after DMACA staining and embedding in LR White resin. Bars represent 2 mm.

Staining of immature white clover inflorescences with DMACA without fixation or embedding showed that a high level of proanthocyanidins accumulates in flowers (Figure 1A-B). Proanthocyanidin accumulation in flowers was found to be higher in more mature flowers located at the base of developing inflorescences than in less mature terminal flowers (Figure 1B). A high level of proanthocyanidin accumulation was seen throughout petals of mature flowers (Figure 1C). The color of DMACA-stained proanthocyanidin-rich organs and tissues changed from blue to brown/red when white clover inflorescences were embedded in LR White resin (Figure 1D-F). Staining and embedding led to light yellow coloration in all petals and dark brown coloration in carpels of more mature flowers located at the basal parts of immature inflorescences (Figure 1D). The coloration of petals was most intense in mature flowers (Figure 1E-F).

**Figure 2:**
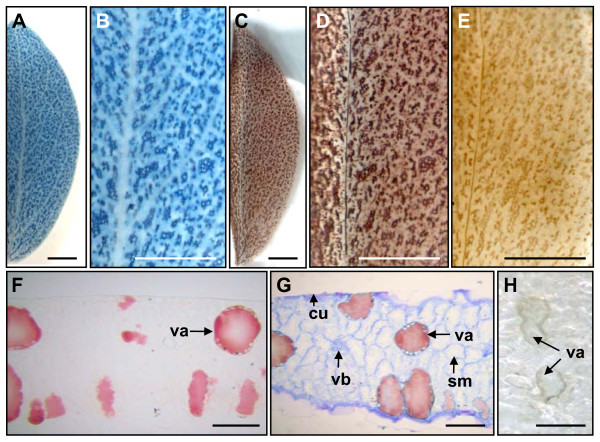
**Proanthocyanidin accumulation in *Lotus corniculatus *leaves**. A *L. corniculatus *leaf stained with DMACA (A-B). A *L. corniculatus *leaf stained with DMACA and embedded in LR White resin (C-D). A *L. corniculatus *leaf embedded in LR White resin without DMACA staining (E). Transverse section of a *L. corniculatus *leaf after DMACA staining and embedding in LR White resin (F-G). In Panel G, the cell walls have been stained with Toluidine Blue after sectioning of the samples shown in panel F. Transverse section of an unstained *L. corniculatus *leaf embedded in LR White resin without DMACA staining (H). cu-cuticle; sm-spongy mesophyll cells; va-vacuole; vb-vascular bundle. Bars represent 200 μm (A-E) and 50 μm (F-H).

*L. corniculatus *and *T. repens *plants showed different spatial patterns of proanthocyanidin accumulation. Fresh leaves of a *L. corniculatus *plant stained with DMACA showed proanthocyanidin accumulation in some mesophyll cells (Figure 2A-B). This was consistent with previous work revealing the presence of proanthocyanidin-containing cells in the mesophyll layer of *L. corniculatus *leaves [28]. Embedding of the DMACA-stained tissues in LR White resin changed the color of proanthocyanidin-accumulating tissues from blue to brown/red (Figure 2C-D). Embedding of unstained *L. corniculatus *leaves in LR White resin resulted in the appearance of pale brown regions corresponding to areas of proanthocyanidin accumulation in DMACA-stained leaves (Figure 2E). An advantage of embedding tissues in a matrix, such as LR White or paraffin, is that physical disruption of tissues and cells is minimal when sectioning is performed. This was clear when proanthocyanidin accumulation was visualized within mesophyll cells of *L. corniculatus *leaves after DMACA staining, embedding in LR White resin and preparation of 10 μm sections. Large, irregularly shaped compartments resembling vacuoles were stained a brown/red color (Figure 2F-G). Staining with Toluidine Blue, a general-purpose alkaline aniline stain commonly used to give a even coloration to the specimen and distinguish it from the embedding material, allowed cell walls to be visualized in leaf tissues (Figure 2G). Light brown coloration was seen in vacuoles of cells when unstained leaf tissues were embedded in LR White resin (Figure 2H). The coloration was most intense near the tonoplast.

**Figure 3:**
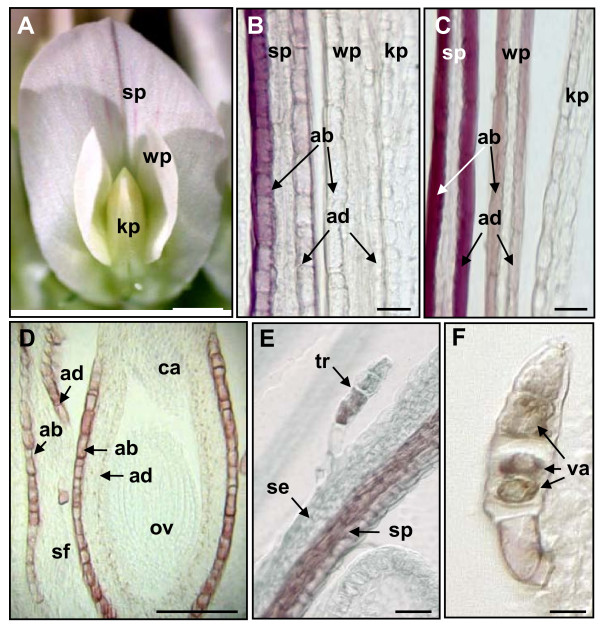
**Cell-specific localization of proanthocyanidins in white clover floral organs**. Corolla of a white clover flower (A). Transverse sections through petals at different stages of development after DMACA staining and embedding in LR White resin (B-C). Longitudinal section through an immature white clover flower (D). Longitudinal section through an immature white clover flower (E). Longitudinal section through a trichome (F). All tissues were stained with DMACA and embedded in LR White resin. ab-abaxial surface of organ; ad-adaxial surface of organ; ca-carpel; kp-keel petals; ov-ovule; se-sepals; sf-stamen filament; sp-standard petal; wp-wing petal; tr-trichomes; va-vacuole. Bars represent 1 mm (A), 50 μm (B-E) and 5 μm (F).

Although histochemical staining of fresh plant organs with DMACA allowed proanthocyanidins to be visualized in white clover plants at a gross level, resolution of fine structure was poor after hand-sectioning. Therefore, embedded tissues were used for cell- and tissue-specific localization of proanthocyanidins in floral organs. Sections of flowers from immature inflorescences stained with DMACA and embedded in LR White resin showed proanthocyanidin accumulation only in glandular trichomes on sepals and in epidermal cells of petals, carpels and stamens (Figure 3). The corolla of a white clover flower is asymmetrical and contains 5 petals: a single large standard petal and two lateral wing petals, which enclose two interior keel petals (Figure 3A). Proanthocyanidins and/or their monomers were first observed in the epidermal layer of standard petals and progressively accumulated in the epidermal layer of the wing and keel petals, respectively (Figure 3B-C). High-resolution imaging revealed an asymmetric pattern of proanthocyanidin accumulation within the epidermal layer, starting on the abaxial surface and proceeding to the adaxial surface of petals (Figure 3B-C).

When immature flowers were stained with DMACA and embedded in LR White resin prior to sectioning, brown/red coloration was most pronounced in the epidermal cell layer on the abaxial and adaxial surfaces of stamen filaments and on the abaxial surface of carpels (Figure 3D). A longitudinal section of an immature flower showed the presence of brown/red coloration in a standard petal and a trichome (Figure 3E). Brown coloration was seen in vacuoles of multiple cells within a trichome when viewed under higher magnification (Figure 3F).

**Figure 4:**
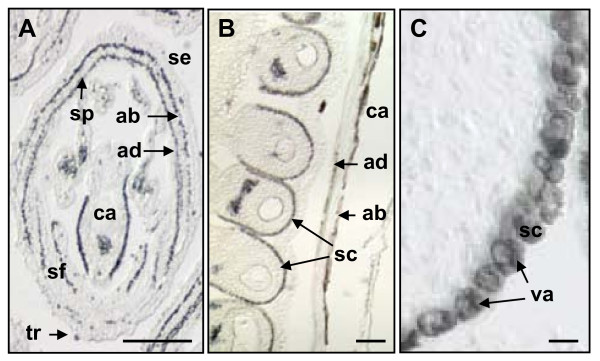
**Accumulation of a colored metabolite in epidermal cells of white clover floral organs**. Longitudinal section through an immature white clover flower (A), and developing seeds in a mature flower (B, C) after embedding of tissues in paraffin. ab-abaxial surface of organ; ad-adaxial surface of organ; ca-carpel; sc-seed coat; se-sepal; sf-stamen filament; sp-standard petal; va-vacuole. Bars represent 100 μm (A-B) and 10 μm (C).

Brown coloration of proanthocyanidin-accumulating cells also resulted from embedding of unstained white clover floral tissues in a paraffin matrix. The resolution of paraffin sections was not as good as LR White sections, but still allowed tissue-specific localization of proanthocyanidins. Figure 4 shows high magnification images of 8 μM sections of unstained floral tissues from white clover embedded in paraffin. Cells that corresponded to those stained with DMACA in petals, stamen filaments and carpels exhibited brown to black coloration (Figure 4A). The presence of a brown metabolite in epidermal cells reflected the expected pattern of proanthocyanidin accumulation in virtually all epidermal cells located on the abaxial and adaxial surfaces of the petals and most epidermal cells on the abaxial surfaces of carpels and stamen filaments. Figure 4B shows accumulation of the brown metabolite in developing seed coats in mature flowers. Figure 4C shows seed coat cells under higher magnification with brown coloration visible in vacuoles. Embedding of DMACA-stained tissues in paraffin changed the color of DMACA stained tissues from blue to brown and this was difficult to distinguish from the brown coloration that appeared in proanthocyanidin-rich tissues as a result of the embedding process.

The brown metabolite is likely to be oxidized proanthocyanidins for two main reasons. The spatio-temporal pattern of the metabolite correlated well with DMACA staining of cells in developing white clover flowers. Non-enzymatic oxidation of proanthocyanidins could lead to the formation of quinoidal compounds. Semiquinones and quinones are highly reactive species that undergo further non-enzymatic reactions, reacting spontaneously with phenols, amino acids or proteins, yielding a complex mixture of brown products [29].

## Conclusions

A method has been demonstrated for localizing proanthocyanidin deposits in cells of two proanthocyanidin-rich legume species, namely, *Lotus corniculatus *and *Trifolium repens*. Sectioning and microscopic analysis of DMACA-stained tissues embedded in LR White or paraffin matrices allowed high-resolution imaging. The embedding procedure alone allowed the spatial pattern of proanthocyanidin accumulation to be visualized, probably due to the oxidation of proanthocyanidins and flavan-3-ols. This method is simple and allows proanthocyanidins to be visualized in specific tissues and cell types when combined with high resolution microscopic analysis.

## Methods

### 4-Dimethylaminocinnamaldehyde (DMACA) staining and fixing of samples

White clover flowers at different stages of maturity as well as leaves from *Lotus corniculatus *plants were decolorized in absolute ethanol for 3 h and stained for the presence of proanthocyanidins and flavan-3-ols using 0.01% (w/v) 4-dimethylaminocinnamaldehyde (DMACA) in absolute ethanol containing 0.8% w/v hydrochloric acid [5]. Flowers were stained for 20 min and the remaining organs were stained for 2 h before being transferred to 100% ethanol. After DMACA staining, samples were vacuum-infiltrated for 1 min with fixative (6% w/v glutaraldehyde, 4% w/v paraformaldehyde in 50 mM sodium phosphate buffer, pH 7.4) in 1.5 mL microcentrifuge tubes and were incubated for 2 h at 4°C. Samples were then washed three times for 5 min in 50 mM sodium phosphate buffer, pH 7.4.

### Embedding of samples in LR white resin

Samples were dehydrated using 5 sequential 15 min washes in an ethanol series (30%, 60%, 70%, 90% and 100% ethanol). The ethanol was replaced with a 3:7 mixture of LR White resin (ProSciTech, Australia) and absolute ethanol. After 1 h of incubation at ambient temperature, this mixture was replaced with a 7:3 mixture of LR White resin and absolute ethanol and incubated for 1 h at ambient temperature. The samples were subsequently incubated for approximately 14 h at ambient temperature in 100% LR White resin. Finally, samples were placed in plastic capsules containing 100% LR White resin and incubated for approximately 14 h under vacuum at 60°C.

### Embedding of samples in paraffin

Samples were dehydrated using sequential 15 min washes in an ethanol series (30%, 60% and 70%). Embedding of tissues in paraffin was performed as previously described [30,31].

### Sectioning and imaging of embedded samples

Transverse and longitudinal sections of embedded samples in LR White resin (6 - 10 μm) and paraffin (8 μm) were generated using a microtome. Cell walls in some of the sections were stained with 0.05% Toluidine Blue (ProSciTech, Cat. # C078) so that the fine structure of plant tissues could be seen. Images were captured using a Leica MZFLIII light microscope (Leica, Germany) fitted with a CCD camera.

## Competing interests

The authors declare that they have no competing interests.

## Authors' contributions

SWA carried out the lab work, GS, AM and SP conceived of the project. All authors read and approved of the final manuscript.
